# The coordinated development of manufacturing industry and logistics industry in the Yangtze River Economic Belt: Empirical study by stages based on Haken Model

**DOI:** 10.1371/journal.pone.0263565

**Published:** 2022-02-10

**Authors:** Wei Du, Yachen Yang

**Affiliations:** School of Economics and Management, Chongqing University of Posts and Telecommunications, Chongqing, China; Institute for Advanced Sustainability Studies, GERMANY

## Abstract

It has great significance for improving the logistics service ability of the Yangtze River economic belt, optimizing the industrial structure of manufacturing industry, and realizing the integrated development of the Yangtze River economic belt to explore the collaborative evolution of logistics industry and manufacturing industry in the Yangtze River economic belt, and identify the leading position of the collaborative development of the two industries, so as to. Based on the Haken Model, this paper summarizes the coevolution law of logistics industry and manufacturing industry in the Yangtze River economic belt through two-stage empirical analysis, and identifies the order parameters of the co-development of logistics industry and manufacturing industry. The results show that the overall degree of coordination between the logistics industry and the manufacturing industry in the Yangtze River economic belt is high. And the order parameter has been changed from manufacturing industry in 2003–2009 to logistics industry in 2010–2017. The gap between regions has been reduced, and the western region has the advantage of post development.

## 1 Introduction

The Yangtze River economic belt runs across the central region of China, accounting for about half of the country’s GDP. It is one of the regions with the most complete functions, the strongest agglomeration and radiation, and is also the most important force to promote national economic growth [1]. The high-quality development of the manufacturing industry is an inevitable choice for building a modern economic system and promoting a strong manufacturing country. As an important strategy related to China’s overall development, the Yangtze River Economic Belt not only shoulders the mission of leading the country’s high-quality economic development, but also has electronics, steel, manufacturing industry clusters such as textiles and petrochemicals. Therefore, it is of great practical significance to improve the quality of manufacturing industry development in the Yangtze River Economic Belt from the perspective of national strategy or regional economic promotion. As a basic industry, logistics industry is an important link connecting all links of the manufacturing industry of the Yangtze River economic belt. Relying on the natural golden waterway, the Yangtze River economic belt has formed a comprehensive transportation system integrating land transportation, air transportation, water transportation and pipeline transportation. The rich manufacturing industry clusters in the Yangtze River Economic Belt have created a huge market demand for the development of the logistics industry, and the comprehensive transportation system has provided strong support for the development of the manufacturing industry. Manufacturing industry is the lifeblood of China’s national economy, and the cost of the logistics industry is closely related to social and economic efficiency [2]. The mutual promotion between the logistics industry and the manufacturing industry in the Yangtze River Economic Belt can provide useful information for the design of future industrial development strategies [3].

On the whole, the logistics industry and manufacturing industry in the Yangtze River Economic Belt have high efficiency, large scale and good operation [4]. However, in recent years, the development of various regional industries has not synchronized [5], and the degree of industrial transfer within the region has gradually increased. The middle and upper reaches of the western belt undertook the transfer of industries from the eastern and lower reaches, creating a large amount of demand for logistics services during the period, but the development of the logistics industry showed a spatial distribution pattern of "strong east and weak west", which could not effectively support the development of manufacturing industry. Moreover, the co-evolution of the logistics industry and the manufacturing industry’s dominant position affects the distribution of benefits between the two. However, in the current academic research on the coordinated development of the two industries, the dominant position of the two industries is still controversial. From 2003, the manufacturing industry of the Yangtze River economy accounted for about 90% of the total industrial output value. It basically maintained a trend of simultaneous changes with industry and developed active [6], and the Yangtze River Economic Belt has gradually become the strongest and most influential Chinese economy, being one of the regions. After 2010, the Yangtze River Economic Belt has entered a stage of strategic development. During this period, problems such as the same industry structure and regional development imbalance have become increasingly prominent [7], resulting in a slowdown in the economic growth of the Yangtze River Economic Belt. Different from the manufacturing industry, the logistics industry and other productive service industries in the Yangtze River Economic Belt developed slowly in the early stage. With the improvement of related infrastructure and equipment, the scale and rapid growth of the productive service industries such as logistics have gradually increased, and their contribution to the social economy has gradually become prominent.

Since the concept of "Supply-side Structural Reform" was put forward in 2015, all regions have responded positively and comprehensively deepened the adjustment of industrial structure. Facing the arduous task of optimizing, transforming and upgrading the development of manufacturing industry, it has become the only way to realize the interactive and integrated development of manufacturing industry and producer services [8]. The logistics industry is a typical format of the producer service industry. It promotes effective cooperation between the logistics industry and the manufacturing industry, reasonably improves the dynamic matching between the logistics industry and the manufacturing industry, and enables the two industries to interact and develop circularly, which is conducive to strengthening the relationship between the logistics industry and the manufacturing industry and promoting the coordinated development of regional economies. The important position of the Yangtze River Economic Belt in China’s strategic blueprint and the characteristics of its industrial distribution determine the necessity of achieving the coordinated development of logistics and manufacturing in this region [4]. Since the reform and opening up, China has always adopted industrial policies to promote industrial development, and the basic ideas of China’s industrial development can be summarized from the evolution of industrial policies [6]. In recent years, the Chinese government has issued a series of relevant policies to promote the deep integration of modern logistics and manufacturing in the Yangtze River Economic Belt, accelerate the coordinated development of logistics and manufacturing, reduce regional differences, and realize the integrated development of the Yangtze River Economic Belt, thereby driving the overall social economy High-quality operation.

The Yangtze River Economic Belt as an open self-organizing system, which has been affected by multiple factors inside and outside the system in the process of coordinated development and evolution of the logistics industry and manufacturing industry is a complex giant system whose nuture of problem can be grasped based on the theory of regional industrial coordination. Therefore, from the perspective of the overall evolution mechanism of the system, using the self-organization theory and method, this paper constructs the theoretical model of the coordinated development of logistics industry and manufacturing industry in the Yangtze River economic belt, identifies the order parameters of the coordinated development of logistics industry and manufacturing industry in the Yangtze River Economic Belt, and evaluates the synergy level of provinces and cities in the Yangtze River economic belt.

The paper is organized as follows. The first part introduces related literature, sorts out the research results and research status of predecessors, and lays the foundation for the research of this research. The second part expounds the theoretical basis of the coordinated development of logistics and manufacturing in the Yangtze River Economic Belt, and introduces the models used in this article. The third part builds an evaluation index system for the coordinated development of logistics and manufacturing in the Yangtze River Economic Belt, and preprocesses the collected data. The fourth part conducts an empirical test on the data through two stages, and makes an objective explanation. The fifth part draws conclusions and writes policy recommendations.

## 2 Literature review

The coordinated development of industries is one of the hot issues of economic development, and regional economic integration is one of the key directions of regional coordinated development. With the upgrading of the manufacturing industry, the rapid release of logistics demand, the coordination and cooperation between the logistics industry and the manufacturing industry, the trend of integration and interaction has become more obvious, and the coordinated development of logistics and other producer service industries and the manufacturing industry has gradually become a research hotspot for scholars.

The manufacturing industry is the demand basis for the development of the logistics industry. Guerrieri and Lin believe that the manufacturing industry is closely related to the logistics industry and other productive service industries, and emphasized that the manufacturing industry is the basis for the development of the logistics industry, and it is in a supply dominant position in the synergistic relationship between the two [9, 10]. In their research on the impact of the coupling of manufacturing industry and logistics industry on the productivity of manufacturing enterprises, Su pointed out that while the manufacturing industry creates a large amount of demand for logistics services, it also forces the upgrade of logistics services and provides a market foundation for the development of the logistics industry [11]. According to statistics, 70% of the total business volume of the logistics industry is created by the manufacturing industry, and the total logistics value of the manufacturing industry accounts for nearly 90% of the total national logistics value. At the same time, the role of manufacturing industry in promoting the logistics industry is also reflected in the national strategic policies. He believe that the logistics industry is a derivative industry generated by service outsourcing in the process of subdivision and specialization of manufacturing industry, and the development of various strategies of manufacturing industry is an important action program for China’s strategic strength of manufacturing industry, which is of great significance to promote the high-quality development of logistics industry [12].

The logistics industry is an important means for the manufacturing industry to enhance its core competitiveness. Gordon believes that the productive service industry, including logistics, plays a very important role in the development of the manufacturing industry [13]. Moreover, studies by Tranter and Sanders show that the prerequisite for manufacturing industry to increase core competitiveness and productivity is productive services such as logistics, so the logistics industry plays a leading role in the relationship between the two [14, 15]. As specialized supporting activities, productive service industries such as logistics are the link between production and consumption. Research by Fiona et al. found that it can make a positive contribution to manufacturing productivity, and its impact on manufacturing productivity is not limited to local enterprises, but region-oriented [16]. Similarly, the policies related to the logistics industry also have an impact on the manufacturing industry. Arnold et al. found that the policy reform of India’s producer services, especially transportation and telecommunications, has a significant impact on the manufacturing industry, which is an exogenous factor for the development of the manufacturing industry to a certain extent [17]. Without productive services such as logistics, manufacturing cannot occupy the high end of the industrial value chain [18]. At present, China is in the middle and late stage of industry, and according to the practical experience of industrial development in developed countries, producer services such as logistics are an important force in the transformation and upgrading of manufacturing industry [19].

The coordination between the logistics industry and the manufacturing industry is an important way for the logistics industry to improve the level of professional services and the transformation and upgrade the manufacturing industry which enhances its core competitiveness [20]. Since the Second World War, the collaborative relationship between producer services such as logistics and manufacturing has begun to appear. In recent years, with the transformation of the development mode of market economy and the refinement of market division of labor, the dependence between producer services such as logistics and manufacturing has deepened, and the collaborative interaction between them has been deepened day by day. The cost of logistics industry is closely related to social and economic efficiency [2]. Zheng et al. believe that manufacturing industry and logistics industry form a cooperative development relationship based on the core interests of their respective industries and the principle of complementarity. With enterprises as the main body and based on industrial linkage, they provide more efficient logistics services for manufacturing enterprises, reduce the logistics cost of manufacturing industry, improve the core competitiveness and promote their common development [21]. In addition, in the research on the interactive development of China’s marine equipment manufacturing industry and coastal producer services, Xie pointed out that there is a significant mutual support and promotion between the two industries, which in turn will contribute to their own development, so as to form a benign interactive cycle mechanism and promote the development of the two industries in a double helix mode [22]. It also better confirms that with the increasing specialization of social division of labor and the needs of global economic transformation, the productive service industries such as logistics are separated from the manufacturing industry in the form of outsourcing to promote the coordination between the logistics industry and the manufacturing industry, which is conducive to the upgrading of the manufacturing industry and promote the specialization, marketization and socialization of logistics services.

Looking at the existing research, the theoretical system of the coordinated development of logistics industry and manufacturing industry is becoming more and more perfect, and the research methods are becoming more and more abundant, but there are still the following two limitations: First, although the trend of gradual penetration and integration of logistics industry and manufacturing industry is beyond doubt, there are still disputes about their dominant position in the collaborative relationship, and few scholars have studied the differences in the collaborative development level of logistics industry and manufacturing industry in the Yangtze River economic belt. Second, it is possible to study the collaborative development of logistics and manufacturing industry in a phased way, which can grasp the law of the collaborative evolution of the two industries more accurately, but few scholars pay attention to it. Based on the existing research, this paper introduces the Haken Model into the analysis of the collaborative development of manufacturing and logistics industry. From the perspective of industrial collaboration, On the basis of existing research, this paper from the perspective of industrial collaboration, starts from the perspective of the overall evolution of the system, and builds a composite synergistic system of logistics and manufacturing in the Yangtze River Economic Belt based on the Haken model; from the perspective of industrial synergy, identified the sequence parameters of the coordinated system between the logistics industry and the manufacturing industry in the Yangtze River Economic Belt, and the leading position of logistics and manufacturing in the coordinated development is explored. At the same time, this paper analyzes the changes in the level of coordination between the logistics industry and the manufacturing industry in the two phases of the 11 provinces in the Yangtze River Economic Belt, and finds out the synergistic influencing factors to provide a reference for the formulation of relevant policies.

## 3 Theoretical basis and Haken Model

### 3.1 Theoretical basis

#### 3.1.1 Premise of coordinated development of logistics industry and manufacturing industry in the Yangtze River Economic Belt

Logistics industry and manufacturing industry are important parts of the economic system of the Yangtze River economic belt, and their synergy efficiency affects the stable operation and development of the whole economic system [19]. First of all, it is based on the macro level of coordinated development. The manufacturing clusters agglomerated in the Yangtze River Economic Belt provide a huge market demand for the development of the logistics industry, and the logistics industry provides support and guarantees for the manufacturing industry, reducing the operating costs and operating difficulties of the manufacturing industry [11]. When the logistics industry and manufacturing industry develop together, on the one hand, it can increase the total output value of logistics industry and reduce the total cost of social logistics; On the other hand, reduce the logistics cost of manufacturing enterprises, and optimize the industrial structure of manufacturing industry. Secondly, it is based on the micro-level of collaborative development. Promoting the coordinated development of the logistics industry and the manufacturing industry can realize the large-scale, intensive and standardized operation of the logistics industry, and improve the overall service capabilities of the logistics industry. At the same time, the manufacturing industry focuses on core businesses, improves the core competitiveness of products, strengthens competitive advantages, and drives the high-quality operation of the economic system of the Yangtze River Economic Belt [23]. Finally, based on the strategic level of the Yangtze River Economic Belt. The Yangtze River Economic Belt traverses the eastern coast and the central and western inland areas. It realizes the coordination of the division of labor between the logistics industry and the manufacturing industry, promotes overall economic development, and is of extraordinary strategic significance to promote the development of regional economic integration.

The Yangtze River economic belt plays an important strategic role in China’s economic development and is the engine of China’s economic development. Therefore, improving the manufacturing quality and logistics service capacity of the Yangtze River economic belt is very important for social and economic growth [24]. Since China joined the WTO in 2001, the internal and external environment of industrial development has changed greatly, and the international competition is becoming increasingly fierce, which requires manufacturing to improve its core competitiveness. The Yangtze River economic belt as a whole is in the stage of accelerating the process of industrialization, and the manufacturing and industry are changing synchronously with a good development trend. In 2003, the industrial added value was 22588.81 billion yuan (3494.49 billion dollars), accounting for 40.8% of China’s total, and by 2009, it had achieved 172.7% growth and developed rapidly. However, with the economic development entering the new normal, some problems caused by the mechanism need to be solved urgently. Industrial development slowed down from 2010 to 2017. In 2017, the proportion of industrial added value in the country was 47.99%, with a growth rate of 72.7%, so the pulling effect of the Yangtze River Economic Belt on the national economy was relatively weak. The Yangtze River economic belt as a whole is in the late stage of industrialization, and its economic development is gradually dominated by producer services. As a typical business form of producer service industry, logistics industry is also one of the power sources of high-quality economic development [12]. In 2002, the total retail sales of consumer goods in China was 4.0911 trillion yuan (6328.93 billion dollars), which provided a huge demand for the logistics market and brought huge opportunities for the development of the logistics industry. Different from the manufacturing industry, the total post and telecommunications business in the Yangtze River Economic Belt grew steadily from 2003 to 2009, from 2601.22 billion yuan (402.41 billion dollars) to 10,431.07 billion yuan (1613.69 billion dollars). Since 2010, with the continuous promotion of the construction of the Yangtze River economic belt, the infrastructure construction of the logistics industry has been gradually improved, and the modern logistics information platform has been gradually optimized. By 2017, the total postal and telecommunications business of the Yangtze River economic belt had reached 16307.29 billion yuan (25522.74 billion dollars) based on the above data, the development trend of logistics industry and manufacturing industry in the Yangtze River economic belt has changed since 2009. The development speed of manufacturing industry has slowed down since 2010, while the logistics industry has maintained a strong growth momentum. Therefore, in order to clarify the main position of logistics industry and manufacturing industry in the collaborative system of the Yangtze River Economic Belt and the contribution to the coordinated development of logistics industry and manufacturing industry, this paper intends to explore it in two stages from 2003 to 2009 and 2010 to 2017, so as to draw a more scientific research conclusion on the coordinated development of the two industry.

#### 3.1.2 Coordinated development of logistics industry and manufacturing industry

Collaboration aims to explore the system structure and the general principles existing in the process of self-organization of system functions [25]. The theory believes that the overall behavior of the system is determined by the interaction of the internal subsystems, thereby formes a synergistic effect, and makes the sum of the functions of each part smaller than the overall function [26]. In a highly refined market environment, the industry is a complex division of labor network, and industrial coordination is to promote the division of labor between industries, so as to achieve the synergy effect of “1+1>2” between industries [27]. Therefore, the coordination between logistics industry and manufacturing industry is to promote the rational division of labor between them, that is, to cultivate professional third-party logistics enterprises, to accelerate the outsourcing of logistics business in manufacturing industry, to separate logistics business from manufacturing operation, to make the two industries interact with each other, to form a synergy effect, and to realize that the overall synergy function of the two industries is greater than the sum of their respective functions. The cooperation between logistics industry and manufacturing industry requires logistics industry to effectively promote the development of manufacturing industry while improving service quality, realizing market standardization and enhancing professional services. The manufacturing industry stabilizes the core competitiveness of enterprises, focuses on core businesses, divestitures and outsources non-core businesses, and provides a huge market and necessary channels for the growth of the logistics industry.

At the same time, the degree of coordinated development of the logistics industry and the manufacturing industry, as well as the distribution of benefits between the two industry, are closely related to the dominant position of the two industry in the coordinated development [25]. If the logistics industry dominates the evolution of the composite system, the contribution of the logistics industry to the development of the manufacturing industry and the promotion of the collaborative development of the two industry are greater than that of the manufacturing industry. However, the contribution of manufacturing industry to the development of logistics industry, as well as the promoting effect of their collaborative development, are greater than that of logistics industry. For a composite cooperative system, when the order parameter that dominates the evolution of the system changes, it means that the coordinated stage of the system has changed. Therefore, exploring the leading position of the coordinated development of logistics industry and manufacturing industry has a far-reaching impact on promoting the coordinated development of the two industries, reasonably allocating social resources, promoting the optimal layout of industries in the Yangtze River economic belt and realizing the overall optimization.

#### 3.1.3 Self organization condition of logistics and manufacturing industry cooperation

Analyzing from the strategic level of industrial coordinated development, the co-evolution of the logistics industry and the manufacturing industry requires the two subsystems to play their respective comparative advantages, interact with each other, and coordinate the development of the subsystems and the internal development of each subsystem [27]. It is shown in the following three levels. First, in the development of logistics and manufacturing composite system, the free flow of external factors can be realized. The composite system imports basic production materials, advanced science and technology, professional and technical personnel, and industry and social related information from the outside world, and then integrates the internal resources of the system to release products, services, and technologies to the external environment to achieve material, energy and information Exchange [28], so that the composite system gradually enters an ordered state from a disordered state, and evolves from a primary state to an advanced state. At the same time, the logistics industry and the manufacturing industry have different information capabilities, different levels of technology, different levels of talent quality, and different levels of R&D capabilities, leading to system development speeds and development trends that will change over time [29], always in a state of adjustment, is an unbalanced open system. Second, the activities of the two industry complex system include human management factors, the random demand of the market, the random events in the logistics service process, and the uncertainty interference in the manufacturing production process [30], which make the system evolution present diversity and uncertainty, highlighting the characteristics of non-linear change. Thirdly, the evolution of this complex open composite system is nonlinear and non-equilibrium, so random fluctuations are inevitable in the process of co evolution. Whether it is the subtle changes within the industry, the changes in the system structure, or the changes in government policies, the composite system will deviate from the equilibrium state, and form huge fluctuations through the coupling mechanism, which will promote the evolution of the logistics and manufacturing composite system to a new equilibrium state [28].

### 3.2 Co-evolution and the model

#### 3.2.1 Connotation of synergy between logistics industry and manufacturing industry in the Yangtze River Economic Belt

The level of coordination between the logistics industry and the manufacturing industry in the Yangtze River Economic Belt is uneven, and it has not contributed to the integrated development process. With the strengthening of industrial transfer, the development pattern of the logistics industry "strong in the east and weak in the west" cannot effectively support the development of the manufacturing industry. Therefore, promoting the effective coordinated development of the logistics industry and manufacturing industry in the Yangtze River Economic Belt is essential for realizing the integrated development of the Yangtze River Economic Belt and improving the efficiency and benefits of economic operation. By exploring the dominant position in the collaborative relationship between logistics industry and manufacturing industry and analyzing the synergy between logistics industry and manufacturing industry in different time periods in various provinces and cities, their evolution law is obtained, in order to guide the coordinated development of logistics industry and manufacturing industry in the future. German scholar Harken’s synergetics theory pointed out that there are usually only one or several slow variables in complex systems. They control the evolution process of the system, dominate most of the fast variables, determine the structure, function and direction of the evolution of complex systems, and drive the evolution of the system from disorder to order, from low level to high level. Through the microscopic method of synergetics, find the linear instability point and distinguish the fast and slow variables, eliminate the fast variable, and obtain the order parameter equation, which can study the spontaneous formation and evolution process of the ordered structure of the system [31]. The Haken model can be used to describe the structural evolution process that occurs by the interaction of different variables within the system under certain external conditions [32]. At the same time, the complex system composed of logistics industry and manufacturing industry shows the characteristics of openness, non-equilibrium state, nonlinear change and random fluctuation, which conforms to the dissipative structural characteristics of self-organization theory and meets the conditions of self-organization. Among them, the interaction of various variables, both competition and coordination, constitute the driving force of the co-evolution of the system [33]. The collaborative system realizes the synergistic effect under the leading role of order parameters. When the control parameters of logistics subsystem and manufacturing subsystem reach the critical point of the change of composite system state, logistics industry and manufacturing industry begin to interact and produce synergistic effect, so that the two collaborative composite systems realize the orderly structure and then achieve the overall collaborative state. Therefore, based on the Haken Model this paper constructs the evolution model of the collaborative system of logistics and manufacturing in the Yangtze River Economic Belt, identifies the dominant parameters in different time periods in the collaborative system of logistics and manufacturing in the Yangtze River Economic Belt from the perspective of controlling the co evolution variables of the system, analyzes the collaborative situation of various provinces and cities, and obtains the law of their co evolution. And combined with actual situation analysis, pay attention to the management of the order parameters of the coordination system, promote the improvement of the coordination system between the logistics industry and the manufacturing industry in the Yangtze River Economic Belt, and make it develop in a more advanced direction in a coordinated and orderly manner.

#### 3.2.2 The Haken Model

Haken Model can be widely used in the research of different fields by identifying the order parameters of the system to determine the collaborative stage of the system [24]. The Haken model considers a simple motion system consisting of only two subsystems. Which assumed that the subsystem q_1_ is the internal force, which determines the evolution of the entire system, and q_2_ is the subsystem and parameters controlled by the internal force. The dynamic equation is as follows:

$$\frac{dq_{1}}{dt}=-\gamma_{1}q_{1}-\alpha q_{1}q_{2}$$ (1)

$$\frac{dq_{2}}{dt}=-\gamma_{2}q_{2}-\beta q_{1}{}^{2}$$ (2)

According to Eq (2), it can be obtained that when the equation has a definite solution *q*_1_ = *q*_2_ = 0, |*γ*_2_| ≫ |*γ*_1_|, and *γ*_2_ > 0, indicating that the state variable *q*_1_ is the order parameter dominating the evolution of the composite system, q_2_ is the fast decaying variable. This process is called the adiabatic approximation assumption of the simple motion system, and requires that γ_1_ is at least one order of magnitude smaller than γ_2_ [26].

Since γ_1_ can be positive or negative, assuming that it satisfies the adiabatic approximation assumption, let *q*_2_ = 0, then $q_{2}=\frac{\beta }{\gamma_{2}}{q_{1}}^{2}$ therefore solves the system evolution equation:

$$\frac{dq_{1}}{dt}=-\gamma_{1}q_{1}-\;\frac{\alpha \beta }{\gamma_{2}}q_{1}{}^{3}$$ (3)

According to the inverse number integral of the system evolution equation, the potential function can be obtained as:

$$\text{V}\left(\text{q}1\right)=\frac{1}{2}\gamma_{1}q_{1}{}^{2}+\frac{\alpha \beta }{4\gamma_{2}}q_{1}{}^{4}$$ (4)

The potential function has two states:

The first type: when *γ*_1_ > 0, the potential function (4) has a unique solution, at this time q_1_* = 0, the composite system is in a stable state, there will be no interaction between the two subsystems, and no new structure will appear.

The second type: when *γ*_1_ < 0, the solutions of the equation are: q_1_* = 0, $\text{q}1**=\sqrt{\left|\frac{\gamma_{1}\gamma_{2}}{\alpha \beta }\right|},\;\text{q}1***=-\sqrt{\left|\frac{\gamma_{1}\gamma_{2}}{\alpha \beta }\right|},\;$ the system is unstable at this time, q_1_** and q_1_*** are the new non-zero stable solutions of the system, no matter which state the system is in, it will eventually return To the new stable point q_1_** or q_1_***.

The model is discretized as follows;

$$q_{1}\left(\text{t}\right)=\left(1\;-\;\gamma_{1}\right)\;q_{1}\left(\text{t}-\;1\right)-\alpha q_{1}\left(\text{t}-\;1\right)\;q_{2}\left(\text{t}-\;1\right)\;$$ (5)

$$q_{2}\left(\text{t}\right)=\left(1\;-\;\gamma_{2}\right)\;q_{2}\left(\text{t}-\;1\right)-\beta q_{1}{}^{2}\left(\text{t}-\;1\right)$$ (6)

The dynamic evolution behaviors of the system are described as follows:

First, α represents the synergistic influence of *q*_2_ on *q*_1_, and β represents the synergistic influence of *q*_1_ on *q*_2_. When α > 0, *q*_2_ hinders *q*_1_, the greater the absolute value of α the stronger the hindrance; on the contrary, when α < 0, *q*_2_, pushes *q*_1_, and the greater the absolute value of α, the greater the driving force. Similarly, when β > 0, *q*_1_, promotes the growth of *q*_2_, and when β < 0 *q*_1_ hinders the growth of *q*_2_.

Second, *γ*_1_ and *γ*_2_ reflect the ordered state established by the subsystems *q*_1_ and *q*_2_ respectively. *γ*_1_ < 0 means that the subsystem *q*_1_ positively promotes the orderly evolution of the composite system, the larger the absolute value, the higher the order of the system; *γ*_1_ > 0 means that the subsystem *q*_1_ negatively affects the orderly evolution of the composite system, the greater the absolute value is It shows that the higher the degree of disorder in the system. Similarly, *γ*_2_ reflects the influence of subsystem *q*_2_ on the orderly evolution of the composite system. When *γ*_2_ < 0, the subsystem *q*_2_ promotes the increase in the order of the composite system; when *γ*_2_ > 0 the subsystem *q*_2_ presents a negative feedback mechanism that affects the orderly evolution of the composite system.

## 4 Research design

### 4.1 The index system

In order to accurately explore the relationship between the coordinated development of the logistics industry and the manufacturing industry, this paper considers the main aspects reflected by the indicators, which can represent or explain the development of the industry and characterize the degree of order of the system. Based on the industrial structure, organization theory, and related content of industrial development in industrial economics, this paper constructs the evaluation indicators of the logistics industry and manufacturing subsystems from the five dimensions of input, output, scale, structure, and growth. Among them, the input level, output efficiency and structure can reflect the performance of the industry; the industrial structure and the scale of industrial development can reflect the behavior of the industry; and the growth of the industry can represent the development capacity and development trend of the industry. Because the logistics industry and manufacturing industry belong to different industrial types, although the specific indicators of the index layer are different, the development level of the logistics industry and manufacturing industry in the Yangtze River economic belt can be objectively evaluated through the input, output, scale, structure and growth of the system layer, so as to explore the law of their coordinated evolution according to their development level.

Firstly, industrial investment is the sum of human, material and financial resources invested in the development of various industries, and is the basic condition and material basis for industrial development. Considering the availability and integrity of data, the investment in the logistics industry is expressed by the fixed asset investment in the logistics industry, the intensity of fixed asset investment and the number of employees in the logistics industry. Similarly, the investment in manufacturing industry is expressed by the fixed asset investment in manufacturing industry and the intensity of fixed asset investment. Secondly, industrial output is the final result of the production and operation of various industries in a certain period of time, reflecting the development efficiency of the national economy. For the logistics industry, the output value of the logistics industry is used to represent the output of the value form, and the circulation volume per unit output value directly reflects the impact of the development of the logistics industry on commodity circulation. For the manufacturing industry, the contribution rate of total assets, capital preservation and appreciation rate and cost profit rate are typical indicators to measure the economic benefits of the manufacturing industry. Third, the scale of the industry refers to the increase in the number of industries in the development of the industry, and is the basic characterization of the level of industry development. For the logistics industry, the commonly used freight volume, cargo turnover, highway and railway mileage and the total retail sales of social consumer goods are used to characterize the industrial scale. For the manufacturing industry, the number of manufacturing enterprises, the scale of manufacturing assets and the added value of manufacturing industry are used to reflect the scale of manufacturing industry. Fourth, according to the evolution theory of industrial structure, the main problem of industrial structure is the rationalization of industrial structure, that is, various industries coordinate with each other, have strong industrial structure transformation ability, can adapt to the changes of market demand and produce good economic and social benefits [23]. The proportion of manufacturing output value in the secondary industry and the proportion of manufacturing output value in GDP reflect the proportion structure of manufacturing industry in the secondary industry and China’s national economy; The proportion of the output value of logistics industry in the tertiary industry and the proportion of the output value of logistics industry in GDP reflect the proportion structure of logistics industry in the tertiary industry and the whole national economy. Finally, industrial growth refers to the growth level of various industrial sectors of the national economy in terms of input intensity, output efficiency and development scale. It reflects the growth rate, growth power and sustainable development ability of the industry. Based on this, the growth rate of logistics industry is characterized by the growth rate of fixed asset investment, added value, freight volume and freight turnover. Similarly, the growth of manufacturing industry is reflected by the growth rate of fixed asset investment, value-added growth rate, profit growth rate and total asset contribution rate. At the same time, with reference to existing research [8, 23], combined with the scientificity and availability of data, we construct an index system. Which are shown in Tables 1 and 2:

**Table 1:** Evaluation index system of logistics industry.

System layer	Logistics industry indicators	Indicator unit
Input	Fixed asset investment in logistics industry (L1)	100 million yuan
	Fixed assets investment rate of logistics industry (L2)	%
	Number of logistics employees (L3)	people
Output	Total post and telecommunications services (L4)	100 million yuan
	Circulation of unit output value (L5)	yuan
Scale	Highway and railway mileage (L6)	Million kilometers
	Freight volume (L7)	Billion tons
	Cargo turnover (L8)	Billion ton kilometers
	Total retail sales of social consumer goods (L9)	100 million yuan
Structure	The proportion of added value of logistics industry in the added value of tertiary industry (L10)	%
	Proportion of added value of logistics industry in GDP (L11)	%
Growth	Logistics industry fixed asset investment growth rate (L12)	%
	Growth rate of freight volume (L13)	%
	Growth rate of cargo turnover (L14)	%
	Growth rate of added value of logistics industry (L15)	%

**Table 2:** Evaluation index system of manufacturing industry.

System layer	Manufacturing industry indicators	Indicator unit
Input	Fixed Asset Investment in Manufacturing (M1)	100 million yuan
	Fixed asset investment rate of manufacturing industry (M2)	%
Output	Contribution rate of total assets of manufacturing industry (M3)	%
	Manufacturing capital preservation and appreciation rate (M4)	%
	Manufacturing cost profit margin (M5)	%
Scale	Number of manufacturing enterprises (M6)	pieces
	Manufacturing assets scale (M7)	100 million yuan
	Manufacturing value added (M8)	100 million yuan
Structure	Proportion of added value of manufacturing industry in the secondary industry (M9)	%
	Manufacturing value added as a proportion of GDP (M10)	%
Growth	Growth rate of fixed asset investment in manufacturing industry (M11)	%
	Growth rate of profit of manufacturing industry (M12)	%
	Growth rate of total assets contribution of manufacturing industry (M13)	%
	Growth rate of manufacturing value added (M14)	%

### 4.2 Data preprocessing

The data in this article comes from the website of the National Bureau of Statistics of China and the statistical yearbooks of provinces and cities in the Yangtze River Economic Belt, and some indicators are calculated twice from the original data. Due to the lack of some indicators since 2018, and the statistical caliber of some indicators has changed, considering the completeness and unity of the indicators, this article selects the provincial data of logistics and manufacturing in the Yangtze River Economic Belt from 2003 to 2017. Although there is currently no unified and independent data for the logistics industry, China’s transportation, warehousing and postal industries include railway transportation, road transportation, water transportation, air transportation, pipeline transportation, loading, unloading, handling and warehousing, and the postal industry. Except for passenger transportation under road transportation, the rest of the business belongs to the scope of the logistics industry, which bears almost all the functions of the logistics industry, and its added value accounts for about 85% of the total value added of the logistics industry. At the same time, refer to the related results of the research on the relationship between the manufacturing industry and the logistics industry [11, 32], the relevant indicators of the logistics industry in this paper are replaced by transportation, warehousing and postal industries. The proportion of manufacturing industry in industry can reach more than 85%, and the proportion of some large manufacturing provinces can reach more than 90% [3, 21]. Therefore, some index data of manufacturing industry are replaced by industrial data. In order to avoid the influence caused by the disunity of index dimensions in the research, we should first normalize the data, and then entropy weight method can effectively avoid the error caused by subjective factors [34]. Entropy weight method is used to weight each index, and the results are linear weighted sum to obtain the order degree of logistics and manufacturing subsystems.

The specific steps of data preprocessing are as follows: (1) Building a matrix A = (*X*_*ij*_)_*m*×*n*_ (i = 1,2, …, n; j = 1,2, …, m), Since the indicators selected in this article are all positive indicators, we use formula $x_{ij}=\frac{X_{ij}-X_{min}}{X_{max}-X_{min}}$ to normalize the original data, and calculate the proportion of the j-th order parameter $P_{ij}=\frac{x_{ij}}{\sum_{i=1}^{n}x_{ij}}$ (i = 1,2, …, n; j = 1,2, …, m); (2) The entropy of the j-th order parameter is $e_{j}=\text{k}\sum_{i=1}^{n}P_{ij}\text{ln}\;{(P}_{ij})$ (k = lnn, k ≥ 0); (3)The entropy redundancy of the j-th order parameter is *a*_*j*_ = 1-*e*_*j*_(j = 1,2, …, m); (4)The weight of the j-th order parameter is $W_{j}=\frac{a_{j}}{\sum_{j=1}^{m}a_{j}}$ (j = 1,2, …, m; 0 ≤ *W*_*j*_ ≤ 1); (5) The order parameter is linearly weighted to obtain the order degree of the subsystem as $C_{i}=\sum_{j=1}^{m}W_{j}P_{ij}$ (i = 1,2, …, n). The order parameter entropy weight of logistics industry subsystem and manufacturing subsystem is shown in Table 3:

**Table 3:** Order parameter weights of logistics and manufacturing subsystems.

Manufacturing	Order parameter	M1	M2	M3	M4	M5	M6	M7	M8
	Entropy weight	0.1946	0.0685	0.0467	0.0265	0.0384	0.1838	0.1297	0.1289
	Order parameter	M9	M10	M11	M12	M13	M14		
	Entropy weight	0.0115	0.0305	0.0275	0.0445	0.0355	0.0334		
Logistics	Order parameter	L1	L2	L3	L4	L5	L6	L7	L8
	Entropy weight	0.1123	0.0502	0.0919	0.0753	0.0522	0.0752	0.1581	0.1160
	Order parameter	L9	L10	L11	L12	L13	L14	L15	
	Entropy weight	0.0510	0.0551	0.0353	0.0119	0.0237	0.0176	0.0742	

The order parameters of the logistics industry and manufacturing subsystems are linearly weighted to obtain the order degree of the logistics industry and manufacturing subsystems. The data preprocessing results are shown in Tables 4 and 5 (In all tables in this article, Shanghai Province referred to as SH, Jiangsu Province referred to as JS, Zhejiang Province referred to as ZJ, Anhui Province referred to as AH, Jiangxi Province referred to as JX, Hubei Province referred to as HB, Hunan Province is referred to as HN, Chongqing referred to as CQ, Sichuan Province referred to as SC, Guizhou Province is referred to as GZ, Yunnan Province referred to as YN):

**Table 4:** Order degree of manufacturing subsystem.

Years	SH	JS	ZJ	AH	JX	HB	HN	CQ	SC	GZ	YN
2003	0.251 0	0.313 6	0.305 9	0.189 4	0.177 9	0.165 8	0.186 4	0.176 0	0.153 9	0.099 0	0.152 3
2004	0.259 1	0.383 5	0.350 4	0.166 4	0.159 8	0.195 0	0.187 2	0.153 6	0.163 0	0.113 8	0.210 1
2005	0.209 8	0.387 8	0.345 8	0.155 9	0.185 5	0.213 2	0.173 5	0.130 7	0.212 2	0.087 6	0.148 5
2006	0.238 7	0.442 2	0.396 2	0.190 7	0.228 5	0.207 0	0.205 6	0.160 5	0.209 8	0.126 8	0.164 3
2007	0.243 4	0.497 7	0.429 9	0.229 3	0.252 3	0.247 6	0.272 3	0.221 5	0.258 8	0.156 7	0.180 3
2008	0.200 6	0.612 8	0.430 1	0.278 9	0.318 8	0.286 5	0.271 0	0.209 6	0.263 6	0.112 0	0.135 5
2009	0.241 3	0.574 2	0.453 6	0.281 0	0.247 9	0.264 2	0.249 5	0.197 5	0.274 2	0.093 3	0.145 7
2010	0.293 6	0.668 2	0.513 5	0.363 1	0.347 0	0.339 8	0.361 4	0.243 3	0.313 6	0.162 0	0.208 4
2011	0.220 3	0.610 0	0.400 9	0.330 8	0.329 8	0.322 5	0.336 3	0.228 9	0.329 8	0.179 7	0.174 4
2012	0.206 9	0.632 7	0.399 7	0.340 2	0.329 7	0.342 8	0.318 8	0.192 7	0.310 3	0.153 7	0.154 9
2013	0.213 9	0.685 2	0.428 3	0.358 5	0.353 8	0.380 0	0.346 0	0.228 4	0.310 7	0.128 1	0.160 1
2014	0.208 9	0.707 0	0.438 2	0.346 6	0.354 9	0.366 4	0.314 7	0.250 3	0.299 4	0.123 0	0.134 9
2015	0.185 4	0.734 5	0.446 9	0.360 3	0.342 4	0.377 3	0.343 7	0.246 8	0.287 4	0.158 1	0.123 2
2016	0.196 2	0.764 3	0.461 2	0.378 3	0.367 3	0.384 0	0.337 5	0.263 2	0.304 7	0.157 4	0.103 2
2017	0.206 3	0.765 1	0.450 3	0.378 1	0.372 3	0.373 6	0.340 7	0.250 3	0.326 2	0.145 1	0.204 3

**Table 5:** Order degree of logistics subsystem.

Years	SH	JS	ZJ	AH	JX	HB	HN	CQ	SC	GZ	YN
2003	0.221 3	0.193 5	0.193 1	0.175 3	0.219 1	0.231 9	0.181 4	0.150 3	0.167 5	0.154 3	0.187 7
2004	0.201 6	0.203 0	0.169 9	0.161 5	0.202 6	0.202 1	0.162 9	0.148 6	0.158 3	0.116 2	0.146 1
2005	0.240 2	0.219 5	0.198 2	0.158 6	0.168 5	0.179 9	0.167 7	0.144 1	0.162 2	0.136 3	0.156 7
2006	0.266 9	0.232 5	0.226 4	0.168 8	0.176 5	0.220 9	0.188 6	0.154 0	0.187 9	0.165 1	0.172 1
2007	0.294 0	0.244 2	0.223 2	0.166 8	0.154 5	0.215 1	0.196 6	0.153 3	0.189 0	0.176 9	0.156 1
2008	0.275 6	0.260 8	0.228 3	0.222 3	0.172 2	0.233 5	0.223 4	0.169 3	0.225 9	0.195 3	0.114 8
2009	0.270 1	0.290 2	0.266 5	0.213 0	0.168 9	0.250 5	0.268 0	0.180 8	0.256 0	0.208 5	0.153 9
2010	0.306 3	0.325 0	0.297 2	0.222 9	0.176 8	0.253 9	0.271 6	0.173 5	0.271 1	0.220 3	0.188 3
2011	0.316 9	0.350 7	0.319 0	0.261 0	0.187 0	0.293 8	0.296 0	0.215 7	0.294 2	0.225 9	0.175 7
2012	0.335 3	0.380 3	0.347 8	0.297 6	0.208 4	0.317 5	0.310 0	0.220 8	0.324 9	0.235 2	0.166 8
2013	0.313 1	0.430 9	0.355 7	0.353 7	0.216 9	0.351 0	0.310 6	0.247 1	0.345 5	0.250 8	0.210 0
2014	0.350 9	0.467 5	0.384 1	0.374 9	0.236 0	0.370 1	0.324 1	0.257 1	0.411 7	0.258 8	0.221 7
2015	0.398 2	0.466 4	0.425 1	0.335 1	0.222 2	0.383 1	0.328 0	0.263 2	0.404 6	0.260 3	0.226 2
2016	0.396 9	0.478 3	0.439 3	0.372 2	0.243 4	0.431 7	0.355 7	0.290 1	0.457 0	0.271 4	0.273 5
2017	0.439 2	0.525 7	0.490 7	0.377 7	0.225 3	0.431 6	0.357 5	0.297 8	0.477 8	0.288 3	0.323 7

**Fig 1:**
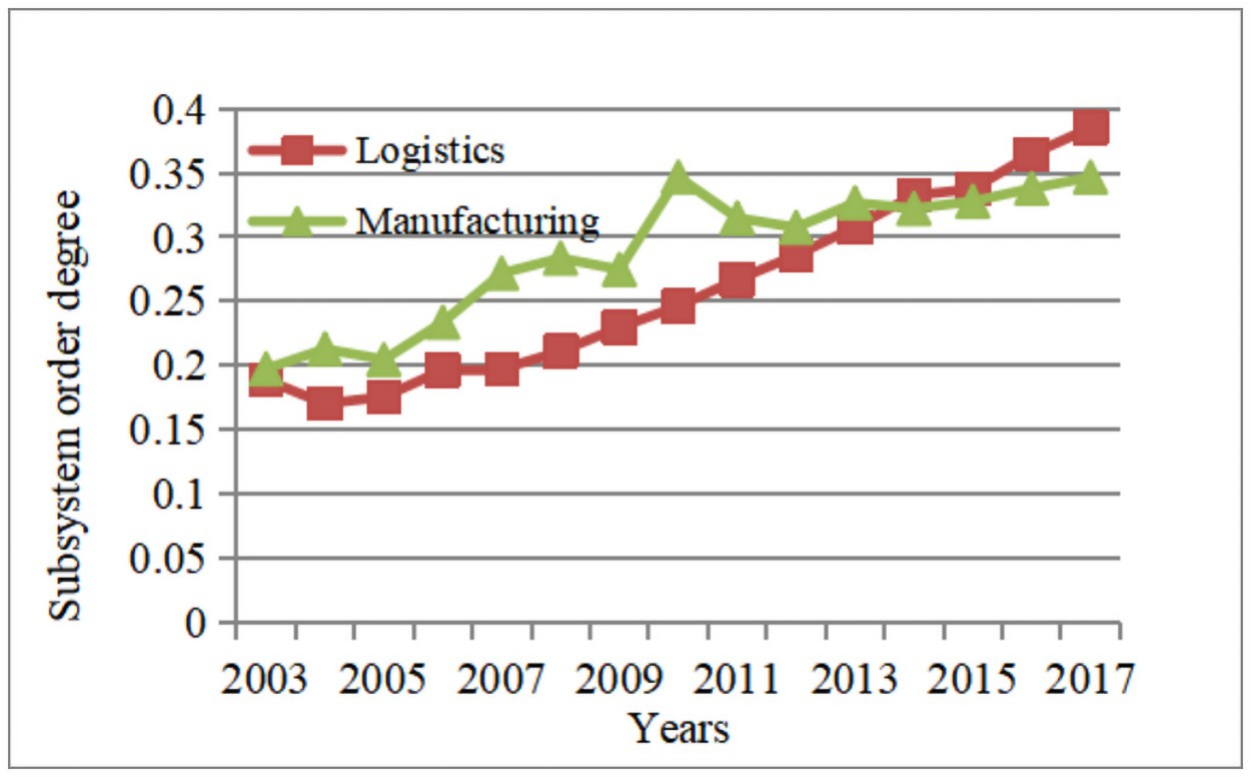
Evolution trend of the order degree of subsystems.

The above calculation results reflect the development of logistics and manufacturing industries in the Yangtze River economic belt from 2003 to 2017. In order to present the evolution law of the order degree of logistics and manufacturing subsystems more intuitively, the average order degree of each subsystem is calculated as shown in Fig 1:

From Fig 1, we can see that the orderliness of the logistics industry subsystem and the manufacturing subsystem of the Yangtze River Economic Belt are both on the rise, and the orderliness of the logistics industry and the manufacturing industry increase alternately. From 2003 to 2009, the orderliness of the manufacturing subsystem of the Yangtze River Economic Belt increased faster than that of the logistics industry; from 2010 to 2017, the orderliness of the manufacturing subsystem of the Yangtze River Economic Belt increased rapidly and gradually slowed down, while the logistics industry The orderliness of the subsystems maintains the original growth rate and grows rapidly.

## 5 Empirical analysis

This paper mainly studies the evolution of the collaborative development of logistics industry and manufacturing industry in the Yangtze River economic belt from 2003 to 2017. Through the identification of order parameters in 2003–2009 and 2010–2017, the change of order parameters of logistics industry and manufacturing industry in different periods is judged, and the synergy level of provinces in the Yangtze River economic belt is evaluated according to the collaborative score. Using the econometric software eviews 10.0, this paper empirically tests the model equation with dynamic panel data regression.

### 5.1 The first stage (2003–2009) order parameter identification

#### 5.1.1 Building model

(1) Assuming that the ordering degree M of the manufacturing subsystem of the Yangtze River Economic Belt is the order parameter of the composite system, and the ordering degree L of the logistics industry subsystem is a fast variable, that is, *q*_1_ = *M*, *q*_2_ = *L*, then the equation of motion is:

$$\text{M}\left(\text{t}\right)=\underset{\left(5.770\;660\right)}{1.001\;004\;\text{M}\left(\text{t}-1\right)}-\underset{\left(0.195\;357\right)}{0.125\;267\;\text{M}\left(\text{t}-1\right)}\;\text{L}\underset{R_{1}^{2}=0.891\;6}{\left(\text{t}-1\right)}$$ (7)

$$\text{L}\left(\text{t}\right)=\underset{\left(10.560\;65\right)}{0.870\;053\;\text{L}\left(\text{t}-1\right)}+\underset{\left(2.689\;930\right)}{0.130\;742\;\text{M}\left(\text{t}-1\right)}\;\text{M}\underset{R_{2}^{2}=0.756\;1}{\left(\text{t}-1\right)}$$ (8)

The parameters of the equation are solved with *γ*_1_ = −0.001 004, *γ*_2_ = 0.129 947, α = 0.125 267, β = 0.130 742, and the goodness of fit of the two equations is $R_{1}^{2}=0.891\;6,\;R_{2}^{2}=0.7565$ the fitting degree is good, and the regression effect is significant. At this time, *γ*_2_ > |*γ*_1_|, *γ*_2_ ≫ *γ*_1_ satisfies the adiabatic approximation hypothesis, and the model assumption (1) is established, that is L changes faster than M, so the order degree M of manufacturing subsystem is the order parameter of the leading composite system coevolution with small damping and slow attenuation [26].

(2) Assuming that the ordering degree L of the logistics industry subsystem of the Yangtze River Economic Belt is the order parameter of the composite system, and the ordering degree M of the manufacturing subsystem is a fast variable, that is, *q*_1_ = *L*, *q*_2_ = *M*, then the equation of motion is:

$$\text{L}\left(\text{t}\right)=\underset{\left(7.449\;503\right)}{0.760\;689\;\text{L}\left(\text{t}-1\right)}-\underset{\left(3.002\;66\right)}{0.419\;50\;\text{L}\left(\text{t}-1\right)}\;\text{M}\underset{R_{3}^{2}=0.762\;1}{\left(\text{t}-1\right)}$$ (9)

$$\text{M}\left(\text{t}\right)=\underset{\left(19.350\;09\right)}{1.032\;23\;\text{M}\left(\text{t}-1\right)}+\underset{\left(0.052\;068\right)}{0.018\;69\;\text{L}\left(\text{t}-1\right)}\;\text{L}\underset{R_{4}^{2}=0.891\;5}{\left(\text{t}-1\right)}$$ (10)

The parameters of the equation are solved *γ*_1_ = 0.239 311, *γ*_2_ = −0.032 23, α = 0.419 5, β = 0.018 69. At this time, *γ*_2_ is negative and *γ*_2_ ≪ *γ*_1_, which does not satisfy the adiabatic approximation assumption, so the model assumption (2) does not hold.

#### 5.1.2 Solving potential function

Assumption (1) is established, further analyze the evolution behavior of the composite system of the logistics industry and the manufacturing industry, judge the state of the system, and according to the value of the solved equation parameters, enter the Eq (4) to obtain the system evolution equation as:

$$\text{dM}/\text{dt}=\frac{1}{1\;000}M-\frac{63}{500}M^{3}$$ (11)

The potential function of the system can be obtained by continuing to solve the inverse integral of the system evolution equation as follows:

$$\text{VM}=-\frac{1}{2\;000}M^{2}+\frac{63}{2\;000}M^{4}$$ (12)

The order degree values of the manufacturing subsystem are all greater than 0, so the image of the potential function only considers the part of M>0. Let $\frac{\text{dM}}{\text{dt}}=0$, the three solutions of the potential function are: M* = 0, M** = 0.089, M*** = −0.089 3. According to the solution of the potential function, the stable point of the composite system is A(0.0893, -0.000 002), When the synergy value is 0.0893, the composite system reaches equilibrium and is in a highly synergistic state.

#### 5.1.3 Synergy evaluation

The distance d between any point B in the logistics and manufacturing composite system of the Yangtze River Economic Belt and the stable point A of the composite system determines the state of this point B. Therefore, the status evaluation function of the composite system is:

$$\text{d}=\sqrt{{\left(M-0.089\;3\right)}^{2}+{\left(V\left(M\right)+0.000\;002\right)}^{2}}$$ (13)

When the value of d is less than or equal to the stable solution of the system, the smaller the value of d, the higher the degree of synergy of the composite system; and the larger the value of d, the lower the degree of synergy of the composite system. When the value of d is greater than the stable solution of the system, it indicates that the system jumps to a new cooperative state. The 2003–2009 composite system collaborative score value is shown in Table 6:

**Table 6:** Scores for the coordinated development of the Yangtze River Economic Belt in the first stage.

Years	SH	JS	ZJ	AH	JX	HB	HN	CQ	SC	GZ	YN
2003	0.161 7	0.224 3	0.216 6	0.100 1	0.088 6	0.076 5	0.097 1	0.086 7	0.064 6	0.009 7	0.063 0
2004	0.169 8	0.294 2	0.261 1	0.077 1	0.070 5	0.105 7	0.097 9	0.064 3	0.073 7	0.024 5	0.120 8
2005	0.120 5	0.298 5	0.256 5	0.066 6	0.096 2	0.123 9	0.084 2	0.041 4	0.122 9	0.001 7	0.059 2
2006	0.149 4	0.352 9	0.306 9	0.101 4	0.139 2	0.117 7	0.116 3	0.071 2	0.120 5	0.037 5	0.075 0
2007	0.154 1	0.408 4	0.340 6	0.140 0	0.163 0	0.158 3	0.183 0	0.132 2	0.169 5	0.067 4	0.091 0
2008	0.111 3	0.523 5	0.340 8	0.189 6	0.229 5	0.197 2	0.181 7	0.120 3	0.174 3	0.022 7	0.0462
2009	0.152 0	0.484 9	0.364 3	0.191 7	0.158 6	0.174 9	0.160 2	0.108 2	0.184 9	0.004 0	0.056 4
Means	0.145 6	0.369 5	0.298 1	0.123 8	0.1351	0.136 3	0.131 5	0.089 2	0.130 0	0.023 9	0.073 1

Comparing the scores and mean values of different time points in Table 6, it is found that the collaborative scores in 2006 are similar to the mean values, and the collaborative scores after 2006 are almost higher than the mean values, which means that the overall composite collaborative system will continue to develop and evolve to a higher level of collaborative development after entering a new collaborative state. According to the calculation results in Table 4, in 2009, the order degree of manufacturing enterprises in the Yangtze River Economic Belt exceeded the system stable solution by 0.089 3, and the order degrees of Jiangsu and Zhejiang reached 0.57 and 0.49, which were far greater than the system stable solution. Shanghai, Anhui, Jiangxi, Hubei, Hunan and Sichuan concentrated in the middle of 0.2–0.3, while Chongqing, Guizhou and Yunnan were below 0.2, showing a ladder distribution.

The results of order parameter identification show that manufacturing dominates the evolution process of collaborative system in the first stage (2003–2009). The order of manufacturing industry is considered by the 14 indicators in Table 2. In combination with Table 3, the investment weight of fixed assets is 19.46%, and the weight of the 3 indicators describing the scale is more than 12.89%, which is greater than the weight of other indicators. Therefore, the coordinated development of the Yangtze River Economic Belt should increase investment in fixed assets in the manufacturing industry in each region, and pay attention to the scale of investment, the speed of investment, and the relationship between investment proportions. In addition, all provinces and cities should also actively guide and provide policy support to expand the scale of manufacturing industry, in which the number of enterprises can reflect the regional economic vitality and marketization to a certain extent, and make contributions to the overall highly orderly and coordinated development of the Yangtze River Economic Belt.

The model assumption (1) is established, where α = 0.125 267 is positive, indicating that the order of logistics industry in the Yangtze River economic belt has a negative impact on the order of manufacturing industry in 2003–2009. The orderliness of the logistics industry is measured by the 15 indicators in Table 3. Combined with Table 3, the weight of the 2 dimensions of investment and scale is 65.47%, and the weight of growth is 12.74%. This shows that in this stage of 2003–2009, the investment in infrastructure construction of the logistics industry in the Yangtze River economic belt cannot keep up with the demand of the development of the manufacturing industry, and the logistics industry cannot effectively support and promote the development of the manufacturing industry, It did not play a positive supporting role in the coordinated development of the two. In addition, β = 0.130 742 is positive, indicating that the manufacturing industry of the Yangtze River economic belt has a positive role in promoting the development of logistics industry in 2003–2009. Combined with Table 3, the weight of investment and scale of manufacturing industry exceeds 70%, which means that while the manufacturing industry self-development in 2003–2009, the development of animal flow industry has made positive contributions to the coordinated development of logistics industry and manufacturing industry in the Yangtze River economic belt.

### 5.2 The second stage (2010–2017) order parameter identification

#### 5.2.1 Building model

(1) Assuming that the order degree M of the manufacturing sub system of the Yangtze River economic belt is the order parameter of the composite system, and the order degree L of the logistics sub system is the fast variable of the system, that is, the motion equation is:

$$\text{M}\left(\text{t}\right)=\underset{\left(13.107\;02\right)}{0.842\;117\;\text{M}\left(\text{t}-1\right)}-\underset{\left(2.697\;068\right)}{0.352\;054\;\text{M}\left(\text{t}-1\right)}\text{L}\underset{R_{1}^{2}=0.967\;1}{\left(\text{t}-1\right)}$$ (14)

$$\text{L}\left(\text{t}\right)=\underset{\left(28.414\;80\right)}{1.015\;228\;\text{L}\left(\text{t}-1\right)}+\underset{\left(0.877\;864\right)}{0.020\;144\;\text{M}\left(\text{t}-1\right)}\text{M}\underset{R_{2}^{2}=0.943\;3}{\left(\text{t}-1\right)}$$ (15)

The parameters of the equation are solved with *γ*_1_ = 0.157 833, *γ*_2_ = −0.015 228, α = 0.353 054, β = 0.020 144. Moreover, *γ*_2_ is negative and *γ*_2_ ≪ *γ*_2_, which does not satisfy the assumption of the insulation approximation, so the model assumption (1) is not valid.

(2) Assuming that the order degree L of the logistics sub system in the Yangtze River economic belt is the order parameter of the composite system, and the order degree M of the manufacturing sub system is a fast variable, *q*_1_ = *L*, *q*_2_ = *M*, the motion equation is:

$$\text{L}\left(\text{t}\right)=\underset{\left(21.242\;51\right)}{1.005\;228\;\text{L}\left(\text{t}-1\right)}-\underset{\left(0.757\;082\right)}{0.039\;272\;\text{L}\left(\text{t}-1\right)}\text{M}\underset{R_{1}^{2}=0.943}{\left(\text{t}-1\right)}$$ (16)

$$\text{M}\left(\text{t}\right)=\underset{\left(35.158\;64\right)}{0.967\;467\;\text{M}\left(\text{t}-1\right)}+\underset{\left(2.237\;215\right)}{0.178\;848\;\text{L}\left(\text{t}-1\right)}\text{L}\underset{R_{2}^{2}=0.966\;2}{\left(\text{t}-1\right)}$$ (17)

The parameters of the equation are solved with *γ*_1_ = −0.005 228, *γ*_2_ = 0.032 533, α = 0.039 272, β = 0.178 848, and the goodness of fit of the two equations are $R_{1}^{2}=0.943\;1,\;R_{2}^{2}=0.966\;2$ the fit is good, and the regression effect is significant. At this time *γ*_2_ > |*γ*_1_|, *γ*_2_ ≫ *γ*_1_ satisfy the insulation approximation assumption, and the model assumption is established. Therefore, M changes faster than L, and the order degree L of the logistics industry subsystem is an order parameter with small damp and slow decline, which dominates the collaborative evolution of the composite collaborative system.

#### 5.2.2 Solving potential function

Assuming (2) is true, further analyze the evolution behavior of the composite cooperative system and judge the state of the system. According to the values of the solved equation parameters, the system evolution equation is obtained by introducing Eq (4) as:

$$\text{dL}/\text{dt}=\frac{13}{2\;500}L-\frac{1\;081}{5\;000}L^{3}$$ (18)

The potential function of the system can be obtained by continuing to solve the inverse integral of the system evolution equation as follows:

$$\text{V}(\text{L})=-\frac{13}{5\;000}L^{2}+\frac{1\;081}{20\;000}L^{4}$$ (19)

The order values of the logistics subsystems are all greater than 0, so the potential function only considers the part with L>0. Let $\frac{\text{dL}}{\text{dt}}=0$, The solution to the potential function are: L* = 0, L** = 0.155 1, L*** = −0.155 1. According to the solution of the potential function, the stable point of the composite system is A (0.155 1, -0.000 03). When the synergy value is 0.155 1, the composite system reaches equilibrium and is in a highly coordinated state.

#### 5.2.3 Synergy evaluation

The distance d between any point B in the logistics and manufacturing composite system of the Yangtze River Economic Belt and the stable point A of the composite system determines the state of this point B. Therefore, the status evaluation function of the composite system is:

$$\text{d}=\sqrt{{\left(L-0.155\;1\right)}^{2}+{\left(V\left(L\right)+0.000\;03\right)}^{2}}$$ (20)

When the value of d is less than or equal to the stable solution of the system, the smaller the value of d, the higher the degree of synergy of the composite system; and the larger the value of d, the lower the degree of synergy of the composite system. When the value of d is greater than the stable solution of the system, it indicates that the system jumps to a new cooperative state. The 2010–2017 composite system collaborative scores value is shown in Table 7:

**Table 7:** Scores for the coordination of the Yangtze River Economic Belt in the second stage.

Years	SH	JS	ZJ	AH	JX	HB	HN	CQ	SC	GZ	YN
2010	0.151 2	0.169 9	0.142 1	0.067 8	0.021 7	0.098 8	0.116 5	0.018 4	0.116 0	0.065 2	0.033 2
2011	0.161 8	0.195 6	0.163 9	0.105 9	0.031 9	0.138 7	0.140 9	0.060 6	0.139 1	0.070 8	0.020 6
2012	0.180 2	0.225 2	0.192 7	0.142 5	0.053 3	0.162 4	0.154 9	0.065 7	0.169 8	0.080 1	0.011 7
2013	0.158 0	0.275 8	0.200 6	0.198 6	0.061 8	0.195 9	0.155 5	0.092 0	0.190 4	0.095 7	0.054 9
2014	0.195 8	0.312 4	0.229 0	0.219 8	0.080 9	0.215 0	0.169 0	0.102 0	0.256 6	0.103 7	0.066 6
2015	0.243 1	0.311 3	0.270 0	0.180 0	0.067 1	0.228 0	0.172 9	0.108 1	0.249 5	0.105 2	0.071 1
2016	0.241 8	0.323 2	0.284 2	0.217 1	0.088 3	0.276 6	0.200 6	0.135 0	0.301 9	0.116 3	0.118 4
2017	0.284 1	0.370 6	0.335 6	0.222 6	0.070 2	0.276 5	0.202 4	0.142 7	0.322 7	0.133 2	0.168 6
Means	0.202 0	0.273 0	0.227 3	0.169 3	0.059 4	0.199 0	0.164 1	0.090 6	0.218 3	0.096 3	0.068 1

Comparing the scores and mean values of different time points in Table 7, it is found that the scores and mean values of each province from 2013 to 2014 are similar, and the collaborative scores are higher than the mean values after 2014, and in an upward trend. Logistics industry and manufacturing industry continue to evolve in coordination, and there is still room for coordinated development. According to the calculation results in Table 5, the order degree of logistics subsystems exceeded the stable solution of the system by 0.1551 in 2017, of which Shanghai, Jiangsu, Zhejiang, Hubei and Sichuan were more than 0.4, Anhui, Hunan and Yunnan were between 0.3–0.4, Jiangxi, Chongqing and Guizhou were between 0.22–0.3. It means that the Yangtze River Economic Belt has entered the evolutionary process of leaping to a higher level of coordinated development, and the tiered nature of various regions has relatively weakened.

The results show that the logistics industry dominates the evolution process of collaborative system in the second stage (2010–2017). The order degree of logistics industry subsystem is considered by 15 indicators in Table 1. Combined with Table 3, the index weight of input dimension is 25.44%, and the weight of freight volume and freight turnover volume is 27.41%, which is a relatively large weight. Therefore, the logistics industry and manufacturing industry in the Yangtze River Economic Belt will develop in a coordinated way. Local governments should pay attention to increasing the investment in fixed assets of the logistics industry and increasing the introduction of logistics professionals, from 2010 to 2017. In addition, the foreign freight volume and freight turnover can reflect the development level of the logistics industry from the side. The local relevant institutions should pay attention to improving the freight management level and freight technology level, so as to comprehensively improve the development level of the logistics industry and realize the highly coordinated development of the two industries in the Yangtze River Economic Belt.

The model hypothesis (2) is established, where α = 0.039 272 is positive, indicating that the manufacturing industry in the Yangtze River Economic Belt from 2010 to 2017 did not significantly promote the logistics industry and did not have a positive impact on the coordinated development of the two industries. In addition, β = 0.178 848 is positive, indicating that the development of the logistics industry in 2010–2017 has a positive impact on the development of the manufacturing industry. Combined with Table 3, the input weight of the logistics industry is 25.44%, the output weight is 12.75%, the scale weight is 34.93%, and the structural dimension weight is 9.04%, showing that the logistics industry can promote the development of manufacturing industry by increasing investment and expanding scale.

In order to show the evolution relationship between the collaborative score of the composite system and the ordering degree of the sub-system in detail, the average of the ordering degree of the logistics and manufacturing sub-systems and the average of the collaborative degree of the composite system are calculated, as shown in Table 8:

**Table 8:** 2003–2017 mean values of order degree of subsystems and coordination value of composite system.

Years	2003	2004	2005	2006	2007	2008	2009	
Composite system	0.108	0.124	0.116	0.144	0.183	0.194	0.185	
Logistics	0.189	0.170	0.176	0.196	0.197	0.211	0.230	
Manufacturing	0.197	0.213	0.205	0.234	0.272	0.284	0.275	
Years	2010	2011	2012	2013	2014	2015	2016	2017
Composite system	0.091	0.112	0.131	0.153	0.177	0.182	0.209	0.230
Logistics	0.246	0.267	0.286	0.308	0.332	0.337	0.364	0.385
Manufacturing	0.347	0.315	0.307	0.327	0.322	0.328	0.338	0.347

**Fig 2:**
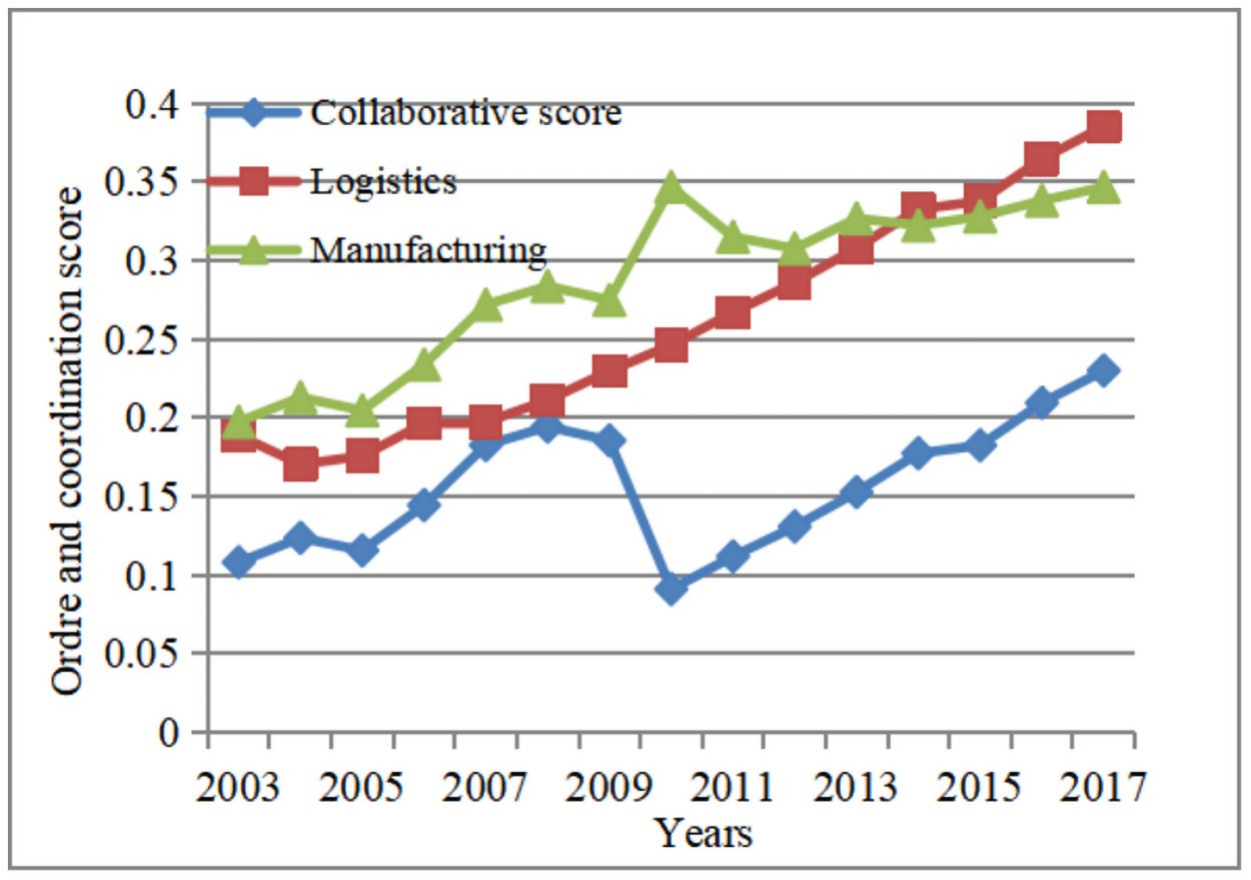
Evolution trend of order degree and cooperative score.

Because the score of the two stages exceeds the critical point of the system, both stages enter a higher level of collaboration. As showed as Fig 2, the synergy level of logistics industry and manufacturing industry in the Yangtze River Economic Belt showed an upward trend from 2003 to 2017. The collaborative score of composite system changes synchronously with the order degree of manufacturing subsystem from 2003 to 2009, and changes synchronously with the order degree of logistics subsystem from 2010 to 2017 which conforms to the evolution law of two-stage order parameters. Combining the model parameters, the role of the manufacturing industry in promoting the logistics industry from 2003 to 2009 is greater than that of the logistics industry in promoting the manufacturing industry; and the role of the logistics industry in promoting the manufacturing industry from 2010 to 2017 is greater than the role of the manufacturing industry in promoting the logistics industry. At the lowest point of collaborative scores, the order degree of manufacturing subsystem increases sharply, and the order degree of logistics subsystem increases steadily, which indicates that the improvement of synergy level of composite system is not due to the development of a subsystem [26], but only when two industries develop simultaneously, the economic contribution to the whole society can reach the maximum [16].

## 6 Conclusion and suggestion

### 6.1 Research conclusion

Based on the synergetic theory, this paper uses Haken Model to analyze the co evolution of logistics industry and manufacturing industry in 11 provinces and cities of the Yangtze River Economic Belt in 2003–2009 and 2010–2017, and found the following Analysis conclusion:

1. The coordination system between the logistics industry and the manufacturing industry in the Yangtze River Economic Belt from 2003 to 2009 was dominated by the manufacturing industry. The first stage sequence parameter identification, model assumption (1) results show that the manufacturing industry is the sequence parameter of the coordinated system of logistics and manufacturing in the Yangtze River Economic Belt, leading the evolution of the coordinated system. Moreover, during this period, when the manufacturing industry is the order parameter of the collaborative system, the interaction intensity between the logistics industry and the manufacturing industry in the Yangtze River economic belt is positive, indicating that the logistics industry has not played an effective supporting role in the manufacturing industry and made a positive contribution to the coordinated development of the two industries. On the contrary, the manufacturing industry has positive feedback to the logistics industry, and promotes the co evolution process of the logistics industry and the manufacturing industry. China’s logistics industry started later than western countries. From 2003 to 2009, the manufacturing industry in the Yangtze River economic belt developed actively, but the infrastructure construction of the logistics industry was imperfect, and the marketization level and specialization ability were insufficient to provide integrated solutions for the manufacturing industry. Therefore, during this period, the overall coordination level of the Yangtze River economic logistics industry and manufacturing regions was relatively low.
2. From 2010 to 2017, the coordinated system of logistics and manufacturing in the Yangtze River Economic Belt is dominated by the logistics industry. The second stage sequence parameter identification, model assumption (2) results show that the logistics industry is a coordinated system sequence parameter of the Yangtze River Economic Belt, leading the evolution of the system. Moreover, during this period, when the logistics industry is the order parameter of the collaborative system, the action intensity between the logistics industry and the manufacturing industry is positive, indicating that the promotion effect of the manufacturing industry on the logistics industry is not obvious. On the contrary, the logistics industry plays a positive feedback on the development of the manufacturing industry and makes a positive contribution to the collaborative evolution of the logistics industry and the manufacturing industry. From 2010 to 2017, the logistics industry developed rapidly, investment continued to be stable, infrastructure was gradually improved, scale was further expanded, social and economic benefits gradually became prominent, fully interacted with the manufacturing industry, and the logistics industry and manufacturing industry entered a new stage of coordinated development.
3. The level of coordination among the various regions of the Yangtze River Economic Belt has a significant gradient from 2003 to 2009, and a markedly weakened gradient from 2010 to 2017. The overall coordination between the logistics industry and the manufacturing industry in the Yangtze River Economic Belt is relatively high, but there is still a large room for growth. The change in the leading parameters of the collaborative system indicating that the logistics and manufacturing industries in the Yangtze River Economic Belt have entered a new stage of collaborative evolution. In the first stage from 2003 to 2009, the evolution stage of collaborative development in the eastern region was ahead of that in the central and western regions, and there was a large gap in collaborative scores between the eastern region and the central and western regions. In addition, the collaborative scores of logistics and manufacturing in Shanghai, Yunnan and Guizhou provinces are in a fluctuating downward trend, while the collaborative scores of other provinces show an obvious upward trend, which means that the synergy level of Shanghai, Yunnan and Guizhou provinces tends to be stable from 2003 to 2009, and the other provinces leap forward to a higher stage. In the second stage from 2010 to 2017, the evolution stage of collaborative development in the eastern and lower reaches was ahead of that in the central and western regions, the distribution of collaborative scores was uniform, and the gradient difference was significantly reduced. In addition, the collaborative scores of Anhui Province and Jiangxi Province showed a fluctuating upward trend, and the other provinces increased steadily. The growth rate of the central and western regions is gradually higher than that of the eastern regions, and has maintained a strong growth momentum, with the advantage of late development and catching up.

### 6.2 Suggestions

Based on the above research conclusions, in order to improve the level of coordination between the logistics industry and the manufacturing industry and promote the integrated development of the Yangtze River Economic Belt, the following specific measures are proposed:

1. Accelerate the construction of logistics specialization and integrated services. From the perspective of collaborative development of the logistics industry and manufacturing industry, the Yangtze River Economic Belt should vigorously support the cultivation of third party logistics, achieve professional market operation, encourage the logistics industry to change to modern logistics service providers and supply chain integrators, accelerate the integration construction, actively encourage logistics to enter the manufacturing production lines, deeply understand the operation and management of manufacturing logistics, and establish various forms of cooperation, realize the optimal allocation of resources. At the same time, the "Notice on Accelerating the Implementation of Major Modern Logistics Projects" also clearly stated that the professionalization level of the logistics industry should be further improved.
2. The manufacturing industry actively tries to optimize and integrate business processes. The "Implementation Opinions on Promoting the Deeply Integrated Development of Advanced Manufacturing and Modern Service Industries" proposes to gradually improve the productive services of enterprises and improve the industrial ecology. In the above research, it is found that the interaction between manufacturing industry and logistics industry is insufficient in the Yangtze River economic belt from 2010 to 2017, and the manufacturing industry does not make full use of logistics resources. Therefore, the Yangtze River Economic Belt should actively promote the construction of horizontal integration, separate logistics and other non core businesses from manufacturing industry, strengthen the integration of the two industries, and improve the overall level of coordination.
3. The Yangtze River Economic Belt should promote the flow of regional factors, rationally allocate resources, and achieve integrated development. There is a significant gradient difference in the coordinated development level of logistics industry and manufacturing industry in the Yangtze River Economic Belt. In 2019, Premier Li Keqiang proposed in the government work report: “The development of the Yangtze River Economic Belt must adhere to the coordination of the upper, middle and lower reaches.” Integrated development can improve the efficiency of resource allocation and policy coordination. Each region can not separate its own development from the development of urban agglomerations. It can encourage the gradient transfer and undertaking of industries in river basin cities, realize the free flow of capital, labor, technology and other elements across regions, realize the optimal allocation of resources, and narrow the development differences among regions, Improve the coordination level of the Yangtze River Economic Belt, and then promote the collaborative development level of logistics industry and manufacturing industry.

## Supporting information

S1 File (RAR)
